# Intimate Partner Violence Is Associated with Stress-Related Sleep Disturbance and Poor Sleep Quality during Early Pregnancy

**DOI:** 10.1371/journal.pone.0152199

**Published:** 2016-03-29

**Authors:** Sixto E. Sanchez, Suhayla Islam, Qiu-Yue Zhong, Bizu Gelaye, Michelle A. Williams

**Affiliations:** 1 Universidad Peruana de Ciencias Aplicadas, Lima, Peru; 2 Asociación Civil PROESA, Lima, Peru; 3 Wellesley College, Wellesley, Massachusetts, United States of America; 4 Department of Epidemiology, Harvard T. H. Chan School of Public Health, Boston, Massachusetts, United States of America; University of Alabama at Birmingham, UNITED STATES

## Abstract

**Objectives:**

To examine the associations of Intimate partner violence (IPV) with stress-related sleep disturbance (measured using the Ford Insomnia Response to Stress Test [FIRST]) and poor sleep quality (measured using the Pittsburgh Sleep Quality Index [PSQI]) during early pregnancy.

**Methods:**

This cross-sectional study included 634 pregnant Peruvian women. In-person interviews were conducted in early pregnancy to collect information regarding IPV history, and sleep traits. Adjusted odds ratios (aOR) and 95% confidence intervals (95%CIs) were calculated using logistic regression procedures.

**Results:**

Lifetime IPV was associated with a 1.54-fold increased odds of stress-related sleep disturbance (95% CI: 1.08–2.17) and a 1.93-fold increased odds of poor sleep quality (95% CI: 1.33–2.81). Compared with women experiencing no IPV during lifetime, the aOR (95% CI) for stress-related sleep disturbance associated with each type of IPV were: physical abuse only 1.24 (95% CI: 0.84–1.83), sexual abuse only 3.44 (95%CI: 1.07–11.05), and physical and sexual abuse 2.51 (95% CI: 1.27–4.96). The corresponding aORs (95% CI) for poor sleep quality were: 1.72 (95% CI: 1.13–2.61), 2.82 (95% CI: 0.99–8.03), and 2.50 (95% CI: 1.30–4.81), respectively. Women reporting any IPV in the year prior to pregnancy had increased odds of stress-related sleep disturbance (aOR = 2.07; 95% CI: 1.17–3.67) and poor sleep quality (aOR = 2.27; 95% CI: 1.30–3.97) during pregnancy.

**Conclusion:**

Lifetime and prevalent IPV exposures are associated with stress-related sleep disturbance and poor sleep quality during pregnancy. Our findings suggest that sleep disturbances may be important mechanisms that underlie the lasting adverse effects of IPV on maternal and perinatal health.

## Introduction

Intimate partner violence (IPV), encompassing physical, psychological and sexual abuse, is a serious public health problem that affects women worldwide. The US Centers for Disease Control and Prevention reported that 24.6% of women experience sexual violence (including rape) by an intimate partner during their lifetime, while 15.8% of women experience severe physical abuse by an intimate partner [1]. Globally, IPV prevalence estimates varies from 15% to 71% [2]. A multi-county study indicated that between 20% and 68% of women, aged 15–49 years, reported experiencing sexual and/or physical violence in their lifetime, by a male intimate partner [3]. IPV has also been shown to affect women’s physical and mental health, reduce sexual autonomy and increase the overall risk of unintended pregnancies and multiple abortions [2, 4, 5]. Women who report experiencing IPV during pregnancy have elevated odds of adverse reproductive outcomes, including preeclampsia, abnormal vaginal bleeding and spontaneous abortion or miscarriage [2, 5–8]. Furthermore, pregnant women experiencing IPV have elevated levels of mood and anxiety disorders [9], hyperarousal and chronic stress [10].

Recently, investigators have postulated that neuroendocrine and physiological changes common to pregnancy and parturition as well as social and environmental stressors such as IPV may contribute to reductions in sleep quality and increased risks of insomnia among pregnant women [9, 11, 12]. While there is extensive research pertaining to risks of adverse mental, physical and reproductive health outcomes among women exposed to IPV, few investigators have explored associations of sleep-disturbances and sleep-quality among victims of IPV. Recently, Woods and colleagues reported that victims of IPV experience elevated risks of sleep disturbances, stemming from heightened vigilance and anticipation of violence while asleep [12]. Notably, these observations are consistent with a high incidence of post-traumatic stress disorder and major depression among women who experienced IPV [12, 13]. In the World Health Organization [14] multi-country study on domestic violence, Peru is one of the countries surveyed with a high prevalence of intimate partner violence. Consistent with the WHO report, a recent study conducted among pregnant women in Lima Peru found that the lifetime prevalence of any IPV to be 45.1% [15].Given the high prevalence of IPV and that emerging literature suggest important associations of sleep disturbances with IPV exposure, we used data from a large study of low-income pregnant Peruvian women to assess the extent to which, if at all, maternal IPV experience is associated with stress-related sleep disturbance and poor sleep quality in early pregnancy. An understanding of these relationships is of particular interest given the high burden of gender-based violence and associated adverse mental and physical health outcomes in this population [16, 17]; and opportunities to provide trauma-informed care to affected women during the antepartum period.

## Methods

### Study Population

This cross-sectional study was a part of the Pregnancy Outcomes, Maternal and Infant Study (PrOMIS) Cohort, an ongoing prospective cohort study of pregnant women enrolled in prenatal care clinics at the Instituto Nacional Materno Perinatal in Lima, Peru. The INMP is a referral hospital for maternal and perinatal care in Lima, Peru. The study population for this report is derived from information collected from those participants who enrolled in the PrOMIS Cohort Study between October 2013 and February 2014. The study population included pregnant women who were 18–49 years of age, who spoke and understood Spanish, and who initiated prenatal care prior to 16 weeks gestation. Written informed consent was obtained from all participants. The Institutional Review Boards from the Instituto Nacional Materno Perinatal, Lima, Peru and the Human Research Administration Office at Harvard T.H. Chan School of Public Health, Boston, MA approved all procedures used in this study.

### Analytical Population

During the period 758 eligible women were approached, and 652 (86%) agreed to participate. Eighteen participants were excluded from the present analysis because of missing information concerning experience with abuse in childhood, prevalent IPV and/or information concerning sleep traits. The 18 participants excluded from this analysis did not differ in regards to sociodemographic and lifestyle characteristics as compared with those included. A total of 634 women remained for analysis.

### Data Collection and Variable Specification

Using a structured questionnaire, participants were interviewed by trained research personnel in a private setting. Information regarding maternal socio-demographic and lifestyle characteristics, medical and reproductive history, childhood abuse, intimate partner violence (IPV), and sleep traits was collected. The questionnaire, originally written in English, was translated into Spanish by a team of native Spanish speakers with experience in sleep research. To ensure proper expression and conceptualization of terminologies in local contexts, the translated version was back-translated and modified until the back-translated version was comparable with the original English version

### Intimate Partner Violence Assessment

Questions pertaining to IPV were adapted from the protocol of Demographic Health Survey Questionnaires and Modules: Domestic Violence Module [18] and the World Health Organization (WHO) Multi-Country Study on Violence Against Women [3]. Participants were assessed for a range of physical and/or sexual coercive acts used against them by a current or former spouse or intimate partner without their consent during their life time and 12 months before pregnancy. Women were classified as having experienced moderately severe physical violence if they endorsed any of the following acts: being slapped, having their arms twisted or something thrown at them, being pushed or shoved. Participants were classified as having experienced severe physical violence if they reported experiencing any of the following acts: being hit, kicked, dragged or beaten up, being choked or burnt on purpose, or being threatened or hurt with a weapon (such as, gun, knife, or other object). Participants were classified as having experienced sexual violence if they endorsed any of the following: being physically forced to have sexual intercourse, having had unwanted sexual intercourse because of fear of what the partner might do, and being forced to perform other sexual acts that the respondent found degrading or humiliating. Consistent with the WHO Multi-Country Studies [3], women were categorized as having experienced one or more acts of physical or sexual violence, physical violence only, sexual violence only, or both physical and sexual violence at any time from a current or former male partner during their lifetime and 12 months before pregnancy.

All study personnel were trained on interviewing skills, contents of the questionnaire, and ethical conduct of violence research (including issues of safety and confidentiality). Interviewers were trained to refer participants found to be in physically dangerous situations and/or in immediate need of counseling to psychologists at local women’s organizations, hospital psychiatrists, and battered women’s shelters.

### Vulnerability for Stress-Related Sleep Disturbances

We used the Ford Insomnia Response to Stress Test (FIRST), standardized self-rating questionnaire, to measure the likelihood of the occurrence of sleep disturbance in response to commonly experienced stressors [19]. FIRST has been shown to be a sensitive measure of vulnerability to sleep disturbance and to have a very high test-retest reliability (0.92) [19]. The instrument has been shown to yield valid measures of vulnerability to sleep disturbances in normal non-insomniac individuals using polysomnographic assessment [19]. The instrument includes nine items asking about the likelihood of sleep disruption due to specific stressful situations and more broadly described periods of stress occurring during the day or evening. The possible responses and corresponding score included: not likely = 1, somewhat likely = 2, moderately likely = 3 and very likely = 4. The total score ranges from 9 to 36. High scores on the FIRST indicate greater vulnerability to sleep disruption [20]. Consistent with prior studies, we used the median score (12) to define high and low FIRST score groups [20–24].

### Sleep Quality Assessment

We used the Pittsburgh Sleep Quality Index (PSQI), a 19-item, self-rated questionnaire to assess maternal early pregnancy sleep quality [25]. The PSQI has seven sleep components: sleep duration, disturbance, latency, habitual sleep efficiency, use of sleep medicine, daytime dysfunction due to sleepiness and overall quality of sleep. Each component produced a score ranging from 0 to 3, where a score of 3 indicates the highest level of dysfunction. A global sleep quality score was obtained by summing the individual component scores (range 0 to 21) with higher scores indicative of poorer sleep quality during the previous month. Participants with global scores that exceed 5 were classified as poor sleepers, and those with a score of 5 or less were classified as good sleepers [25]. This classification scheme is consistent with prior studies including those conducted in Peru [26–28].

### Antepartum Depression

The PHQ-9 was used to evaluate maternal antepartum depression [29]. The PHQ-9 is a 9-item questionnaire has a demonstrated reliability and validity for assessing depressive disorders among a diverse group of obstetrics-gynecology patients [30], and in Spanish-speaking Peruvian women [31]. The PHQ-9 instrument asks respondents to rate the relevancy of each statement comprising emotional, cognitive, and functional somatic symptoms over the past two weeks on a four-point scale (a) never; (b) several days; (c) more than half the days; or (d) nearly every day. The PHQ-9 total score is the sum of scores for the nine items for each woman, and ranged from 0–27. We defined presence of antepartum depression based upon total PHQ-9 score, (a) no depressive symptoms (0–9) and (b) antepartum depression (10–27) [29]. A meta-analysis of 14 studies supports the use of a PHQ-9 score of ≥10 to classify subjects with major depressive disorder [32].

### Other Covariates

Participants’ age was categorized as follows: 18–20, 20–29, 30–34, and ≥35 years [16, 17]. Other sociodemographic variables were categorized as follows: educational attainment (≤6, 7–12, and >12 completed years of schooling); maternal ethnicity (Mestizo vs. other); marital status (married or living with partner vs. other); employment status (employed vs. not employed); access to basic foods (hard vs. not very hard); parity (nulliparous vs. multiparous); planned pregnancy (yes vs. no); and early pregnancy body mass index (BMI) (<18.5 kg/m^2^, 18.5–24.9 kg/m^2^, 25–29.9 kg/m^2^, and ≥30 kg/m^2^). Early pregnancy BMI values were based on maternal weight and height measured by trained research personnel.

### Statistical Analysis

Frequency distributions of maternal sociodemographic and reproductive characteristics were examined. Chi-square tests for categorical variables and analysis of variance (ANOVA) for continuous variables were conducted to determine whether there were statistically significant differences in the association between sociodemographic and reproductive characteristics and exposure to any intimate partner violence in the 12 months preceding pregnancy. Multivariate adjusted logistic regression procedures were used to calculate maximum likelihood estimates of odds ratios (ORs) and 95% confidence intervals (CIs) of vulnerability to stress-related sleep disturbance and poor sleep quality, respectively, in relation to IPV exposure. Furthermore, to assess the joint and independent effect of intimate partner abuse and depression on risk of sleep disturbance, we categorized participants into four groups based on combinations of IPV and depression status. The four resulting categories were as follows: (1) no abuse and no depression, (2) abuse only, (3) depression only, and (4) abuse and depression combined. Women with no abuse and no depression comprised the reference group, against which women in the other three categories were compared. We included covariates of *a priori* interest (i.e., maternal age, education, ethnicity, parity and difficulty paying for the basics) in the final multivariate adjusted logistic regression models. All statistical analyses were performed using SAS 9.4 (SAS Institute, Cary, NC, USA). The level of statistical significance was set at *P-value* < 0.05, and all tests were two-sided.

## Results

Sociodemographic and reproductive characteristics of the study population are summarized in Table 1. Approximately 33.0% of study participants reported a history of lifetime exposure to intimate partner violence. Participants who were ever exposed to physical or sexual abuse by an intimate partner, had fewer years of formal education, were more likely to have a difficulty paying for basic needs, and to be nulliparous as compared with those who were not abused. Participants were similar with regards to gestational age at interview.

**Table 1:** Socio-demographic and reproductive characteristics of the study population by types of lifetime intimate partner violence in Lima, Peru (N = 634).

Characteristics	All participants		No physical or sexual abuse		Physical abuse only		Sexual abuse only		Physical and sexual abuse		*P-*value
	(N = 634)		(N = 425)		(N = 148)		(N = 15)		(N = 46)		
	n	%	n	%	n	%	n	%	n	%	
Age (years) ^1^	28.7 ± 6.5		28.4 ± 6.2		29.3 ± 7.2		28.7 ± 7.0		29.9 ± 6.9		0.34
Age (years)											
18–20	19	3.0	11	2.6	4	2.7	1	6.7	3	6.5	0.09
20–29	339	53.5	237	55.8	74	50.0	7	46.7	21	45.7	
30–34	141	22.2	101	23.8	30	20.3	3	20.0	7	15.2	
≥35	135	21.3	76	17.9	40	27.0	4	26.7	15	32.6	
Education (years)											
≤6	26	4.1	14	3.3	9	6.1	1	6.7	2	4.3	0.009
7–12	326	51.4	207	48.7	82	55.4	4	26.7	33	71.7	
>12	281	44.3	203	47.8	57	38.5	10	66.7	11	23.9	
Mestizo ethnicity	472	74.4	318	74.8	113	76.4	12	80.0	29	63.0	0.30
Married/living with a partner	505	79.7	346	81.4	117	79.1	10	66.7	32	69.6	0.19
Employed	315	49.7	204	48.0	78	52.7	11	73.3	22	47.8	0.22
Access to basic foods											
Hard	308	48.6	183	43.1	82	55.4	9	60.0	34	73.9	0.0001
Not very hard	326	51.4	242	56.9	66	44.6	6	40.0	12	26.1	
Nulliparous	294	46.4	223	52.5	51	34.5	7	46.7	13	28.3	0.0002
Planned pregnancy	262	41.3	178	41.9	55	37.2	6	40.0	23	50.0	0.51
Gestational age at interview^1^	9.1 ± 3.6		9.0 ± 3.5		9.3 ± 3.9		8.1 ± 2.8		10.2 ± 3.5		0.12
Early pregnancy body mass index (kg/m^2^)											
<24.9	304	47.9	211	49.6	67	45.3	8	53.3	18	39.2	0.76
25–29.9	233	36.8	150	35.3	57	38.5	6	40.0	20	43.5	
≥30	86	13.6	56	13.2	21	14.2	1	6.7	8	17.4	

Due to missing data, percentages may not add up to 100%.

^1^ mean ± SD (standard deviation)

For continuous variables, *P*-value was calculated using the ANOVA; for categorical variables, *P*-value was calculated using the Chi-square test or the Fisher’s exact test.

### Stress-Related Sleep Disturbances

Table 2 summarizes results from our analysis of early pregnancy stress-related sleep disturbance in relation to maternal history of lifetime and prevalent (12 month prior to the index pregnancy) exposure to IPV. Women with a history of experiencing any abuse by an intimate partner in their lifetime had 1.54-fold increased odds of stress-related sleep disturbances (aOR = 1.54; 95% CI: 1.08–2.17) after adjustment for maternal age, race, parity, and difficulty paying for basics. We assessed the odds of stress-related sleep disturbances according to type of abuse experienced. Compared with women who reported no lifetime history of intimate partner abuse, those who experienced lifetime sexual abuse only (aOR = 3.44; 95% CI: 1.07–11.05) and lifetime physical and sexual abuse (aOR = 2.51; 95% CI: 1.27–4.96) had elevated odds of stress-related sleep disturbances. Associations of stress-related sleep disturbances with maternal exposure to IPV during the past year were generally similar in direction as lifetime IPV (Table 2**, lower panel**). Compared with women who had no IPV in the 12 months prior to pregnancy, those who suffered from any IPV in that time period had a 2.07-fold increased odds of stress-related sleep disturbances (aOR = 2.07; 95% CI: 1.17–3.67).

**Table 2:** Association between intimate partner violence and stress-related sleep disturbance assessed by the Ford Insomnia Response to Stress Test (FIRST-S) during pregnancy (N = 634).

Intimate partner violence	Low FIRST scores ^1^		High FIRST scores ^1^					
	(N = 324)		(N = 310)					
	n	%	n	%	Unadjusted OR (95% CI)		Adjusted OR (95% CI) ^2^	
**Lifetime**								
No abuse	234	72.2	191	61.6	Reference		Reference	
Any abuse	90	27.8	119	38.4	1.62 (1.16, 2.26)		1.54 (1.08, 2.17)	
Types of abuse								
No abuse	234	72.2	191	61.6	Reference		Reference	
Physical abuse only	72	22.2	76	24.5	1.29	(0.89, 1.88)	1.24	(0.84, 1.83)
Sexual abuse only	4	1.2	11	3.5	3.37	(1.06, 10.75)	3.44	(1.07, 11.05)
Physical & sexual abuse	14	4.3	32	10.3	2.80	(1.45, 5.40)	2.51	(1.27, 4.96)
**12 months before pregnancy**								
No abuse	302	93.2	271	87.4	Reference		Reference	
Any abuse	21	6.5	39	12.6	2.07	(1.19, 3.61)	2.07	(1.17, 3.67)
Types of abuse								
No abuse	302	93.2	271	87.4	Reference		Reference	
Physical abuse only	18	5.6	25	8.1	1.55	(0.83, 2.90)	1.60	(0.83, 3.06)
Sexual abuse only	1	0.3	9	2.9	10.03	(1.26, 79.68)	9.94	(1.24, 79.63)
Physical & sexual abuse	2	0.6	5	1.6	2.79	(0.54, 14.48)	2.22	(0.41, 11.96)

Abbreviations: OR, odds ratio; CI, confidence interval

^1^ Low and high FIRST-S scores were defined based on the median score (12) of the FIRST at interview among this study population.

^2^ Adjusted for maternal age (years) at interview, maternal ethnicity (Mestizo vs. other), parity (nulliparous vs. multiparous), and difficulty paying for the very basics (hard vs. not very hard)

We conducted multivariate analyses to assess the independent and joint associations of maternal lifetime IPV exposure and antepartum depression with the odds of stress-related sleep disturbance (Table 3**)**. Women with antepartum depression only (classified as having no lifetime history of IPV) had a 3.82-fold increased odds of stress-related sleep disturbance (aOR = 3.82, 95% CI: 2.27–6.11) as compared with women who had no lifetime history of IPV and no antepartum depression (the referent group). The aOR for women with IPV only (no antepartum depression) was 1.14 (95% CI: 0.75–1.73) when compared with the referent group, and this weak association was not statistically significant. Women with a history of IPV and antepartum depression had a 9.28-fold increased odds of stress-related sleep disturbance (aOR = 9.28; 95% CI: 4.53–19.02) as compared with the referent group. The excess odds of stress-related sleep disturbance associated with a IPV and antepartum depression was greater than the sum of the excess odds for each risk factor considered independently, suggesting a greater-than-additive association of IPV and depression on the odds of stress-related sleep disturbance. The multiplicative interaction term did not reach statistical significance. However, the excess risk of stress-related sleep disturbance associated with abuse and depression was greater than the sum of the excess risk for each risk factor considered independently. For instance for lifetime IPV, there was evidence of a greater-than-additive effect between abuse and depression on the risk of stress-related sleep disturbance (synergy index = 2.79 (95%CI 1.04–7.46), p = 0.04). Similar associations were observed when we considered maternal recent exposure with IPV (**bottom panel of** Table 3).

**Table 3:** Association between intimate partner violence, depression^1^ assessed by the Patient Health Questionnaire-9 (PHQ-9), and stress-related sleep disturbance assessed by the Ford Insomnia Response to Stress Test (FIRST-S) during pregnancy (N = 631).

Intimate partner violence	Low FIRST scores ^2^		High FIRST scores ^2^			
	(N = 323)		(N = 308)			
	n	%	n	%	Unadjusted OR (95% CI)	Adjusted OR (95% CI) ^3^
**Lifetime**						
No abuse, no depression	206	63.8	129	41.9	Reference	Reference
No abuse, depression	27	8.4	60	19.5	3.55 (2.14, 5.88)	3.82 (2.27, 6.44)
Abuse, no depression	80	24.8	61	19.8	1.22 (0.82, 1.82)	1.14 (0.75, 1.73)
Abuse, depression	10	3.1	58	18.8	9.26 (4.57, 18.76)	9.28 (4.53, 19.02)
*P*-value for interaction					0.10	0.11
**12 months before pregnancy**						
No abuse, no depression	268	83.0	171	55.5	Reference	Reference
No abuse, depression	33	10.2	98	31.8	4.65 (3.00, 7.22)	5.10 (3.24, 8.03)
Abuse, no depression	17	5.3	19	6.2	1.75 (0.89, 3.46)	1.90 (0.94, 3.84)
Abuse, depression	4	1.2	20	6.5	7.84 (2.63, 23.31)	7.82 (2.59, 23.65)
*P*-value for interaction					0.95	0.76

Three women were excluded due to missing information on depression.

Abbreviations: OR, odds ratio; CI, confidence interval

^1^ Depression was defined as the PHQ-9 ≥ 10.

^2^ Low and high FIRST-S scores were defined based on the median score (12) of the FIRST at interview among this study population.

^3^ Adjusted for maternal age (years) at interview, maternal ethnicity (Mestizo vs. other), parity (nulliparous vs. multiparous), and difficulty paying for the very basics (hard vs. not very hard)

### Sleep Quality

We next assessed the maternal early pregnancy sleep quality in relation to her lifetime and past year experience with IPV. As shown in Table 4, a lifetime history of any IPV was associated with a 1.93-fold increased odds of poor sleep quality (aOR = 1.93; 95% CI: 1.33–2.81). The odds of poor sleep quality was elevated irrespective of the type of abuse experienced (e.g., physical only, sexual only or both physical and sexual abuse). Similar associations were observed when we considered odds of poor sleep quality in relation to maternal recent experiences with IPV (Table 4**, bottom panel**). The odds of poor sleep quality appeared to be most strongly associated with maternal experience with sexual abuse only (aOR = 10.37; 95% CI: 2.16–49.91) and physical and sexual abuse (aOR = 5.09; 95% CI: 0.95–27.40), although these estimates are statistically unstable as evidenced by the very wide 95% confidence intervals.

**Table 4:** Association between intimate partner violence and sleep quality assessed by the Pittsburgh Sleep Quality Index (PSQI) during pregnancy (N = 634).

Intimate partner violence	Good sleep quality		Poor sleep quality					
	(PSQI > 5) (N = 456)		(PSQI ≤ 5) (N = 178)					
	n	%	n	%	Unadjusted OR (95% CI)		Adjusted OR (95% CI) ^1^	
**Lifetime**								
No abuse	326	71.5	99	55.6	Reference		Reference	
Any abuse	130	28.5	79	44.4	2.00 (1.40, 2.87)		1.93 (1.33, 2.81)	
Types of abuse								
No abuse	326	71.5	99	55.6	Reference		Reference	
Physical abuse only	96	21.1	52	29.2	1.78 (1.19, 2.68)		1.72 (1.13, 2.61)	
Sexual abuse only	8	1.8	7	3.9	2.88 (1.02, 8.15)		2.82 (0.99, 8.03)	
Physical & sexual abuse	26	5.7	20	11.2	2.53 (1.36, 4.73)		2.50 (1.30, 4.81)	
**12 months before pregnancy**								
No abuse	424	93.0	149	83.7	Reference		Reference	
Any abuse	31	6.8	29	16.3	2.66 (1.55, 4.57)		2.27 (1.30, 3.97)	
Types of abuse								
No abuse	424	93.0	149	83.7	Reference		Reference	
Physical abuse only	27	5.9	16	9.0	1.69	(0.88, 3.22)	1.42	(0.72, 2.81)
Sexual abuse only	2	0.4	8	4.5	11.38	(2.39, 54.20)	10.37	(2.16, 49.91)
Physical & sexual abuse	2	0.4	5	2.8	7.11	(1.37, 37.05)	5.09	(0.95, 27.40)

Abbreviations: OR, odds ratio; CI, confidence interval

^**1**^ Adjusted for maternal age (years) at interview, maternal ethnicity (Mestizo vs. other), parity (nulliparous vs. multiparous), and difficulty paying for the very basics (hard vs. not very hard)

We next assessed the independent and joint associations of history of IPV and antepartum depression with the odds of maternal poor sleep quality during early pregnancy. As shown in Table 5 those with antepartum depression (but without lifetime history of IPV) had a 5.69-fold increased odds of poor sleep quality (aOR = 5.69; 95% CI: 3.36–9.64) as compared with women who had no history of IPV and who did not have antepartum depression (the referent group). The aOR for women with a history of IPV only (no antepartum depression) was 1.71 (95% CI 1.04–2.81) when compared with the referent group. Women with a history of IPV and antepartum depression had a 9.36-fold increased odds of poor sleep quality (aOR = 9.36; 95% CI 5.18–16.93). The multiplicative interaction term was not statistically significant (P-value = 0.93) although some evidence of a greater-than-additive effect between abuse and depression on the risk of poor sleep quality was noted (synergy index = 1.55 (95%CI 0.74–3.24), p = 0.04). Associations of similar directions and magnitudes were observed when we considered maternal odds of poor sleep quality in relation to recent experiences with IPV and antepartum depression (Table 5**, bottom panel**).

**Table 5:** Association between intimate partner violence, depression^1^ and stress-related sleep disturbance assessed by the Pittsburgh Sleep Quality Index (PSQI) during pregnancy (N = 631).

Intimate partner violence	Good sleep quality		Poor sleep quality			
	(PSQI > 5) (N = 454)		(PSQI ≤ 5) (N = 177)			
	n	%	n	%	Unadjusted OR (95% CI)	Adjusted OR (95% CI) ^2^
**Lifetime**						
No abuse, no depression	282	62.1	53	29.9	Reference	Reference
No abuse, depression	42	9.3	45	25.4	5.70 (3.41, 9.52)	5.69 (3.36, 9.64)
Abuse, no depression	106	23.3	35	19.8	1.76 (1.09, 2.84)	1.71 (1.04, 2.81)
Abuse, depression	24	5.3	44	24.9	9.75 (5.48, 17.38)	9.36 (5.18, 16.93)
*P*-value for interaction					0.95	0.93
**12 months before pregnancy**						
No abuse, no depression	364	80.2	75	42.4	Reference	Reference
No abuse, depression	58	12.8	73	41.2	6.11 (3.99, 9.34)	6.10 (3.94, 9.44)
Abuse, no depression	23	5.1	13	7.3	2.74 (1.33, 5.66)	2.38 (1.12, 5.06)
Abuse, depression	8	1.8	16	9.0	9.71 (4.01, 23.5)	8.30 (3.36, 20.49)
*P*-value for interaction					0.36	0.36

Three women were excluded due to missing information on depression.

Abbreviations: OR, odds ratio; CI, confidence interval

^1^ Depression was defined as the PHQ-9 ≥ 10.

^2^ Adjusted for maternal age (years) at interview, maternal ethnicity (Mestizo vs. other), parity (nulliparous vs. multiparous), and difficulty paying for the very basics (hard vs. not very hard)

## Discussion

We found that women who experienced IPV in their lifetime were more likely to have stress-related sleep disturbance (aOR = 1.54; 95% CI: 1.08–2.17) and poor sleep quality (aOR = 1.93; 95% CI: 1.33–2.81) as compared with their counterparts who did not experience IPV. Women reporting any IPV in the year prior to pregnancy had increased odds of stress-related sleep disturbance (aOR = 2.07; 95% CI: 1.17–3.67) and poor sleep quality (aOR = 2.27; 95% CI: 1.30–3.97) during pregnancy. These associations were particularly pronounced among women with antepartum depression.

To our knowledge, this is the first study to examine the relationship between maternal IPV victimization and stress-related sleep disturbance during pregnancy. Our results, however, are in general agreement with a number of prior studies that have assessed associations of measures of sleep disturbances and sleep patterns with IPV victimization [10, 33–36]. For example, in an early descriptive study, Saunders reported that 78% of 192 battered women endorsed having trouble sleeping [34]. In another study of IPV victims living in a battered women’s shelter, Humphreys and colleagues [33] found that 82% of participants endorsed having disturbed sleep patterns with frequent night time awakenings as would be seen among individuals with clinically diagnosed sleep disorders. Furthermore, in a study of 609 women in Kentucky, USA, Walker and colleagues noted that victims of IPV (who had protective orders against their abusive partners) reported sleeping an average of 5.7 hours per night (standard deviation = 1.67 hours) [10]. Collectively, these early descriptive studies provide evidence of a high prevalence of sleep disturbances including short sleep duration among victims of IPV. This is particularly concerning given the high prevalence of IPV reported among Peruvian women. A recent study in Peru by Perales et al found the prevalence of physical and sexual violence during pregnancy to be 11.9% and 3.9%, respectively [15]. Our current findings coupled with high burden of IPV in Peru reiterate the need for concerted global health efforts in preventing violence.

A number of analytical epidemiological studies have assessed associations of sleep disturbances with IPV victimization after adjusting for multiple confounding factors. In their study of 208 South Asian women in Greater Boston, Hurwitz et al [36] reported that women who were abused by their current intimate partners, as compared with those who were not abused had a 2.1-fold increased odds of sleep disturbance (95% CI: 1.0–4.2) after multivariate adjustment for recency of immigration and income. This association was recently corroborated by Newton and colleagues [37] who reported a strong statistically significant association of IPV victimization and risk of poor sleep quality. In their study of 199 women recruited from the general community, Newton et al reported that participant history of IPV was associated with a 3.91-fold (95% CI: 1.75–8.73) increased odds of poor sleep quality (as measured using the PSQI) after controlling for stressful life events, vasomotor symptoms, marital status, annual household income and a number of other covariates [37]. Our study findings among pregnant, Peruvian women corroborate these earlier reports and extend the literature by documenting increased odds of stress-related sleep disturbances and poor sleep quality in relation to lifetime and current IPV victimization and type of victimization. Another important point that merits consideration is exposure to IPV leading to developments of post-traumatic stress disorder (PTSD) and depressive disorders of which sleep disturbance is a symptom. Although PTSD was not assessed in the present study, the associations between IPV and sleep disturbances were more pronounced among women with antepartum depression. Woods et al in their study among victims of IPV found that PTSD symptoms statistically significantly associated with subjective sleep quality (β = .33, p <0.01) [12]. Similar finding was reported by other investigators [13]. Taken together, available evidence indicates the long lasting impact of traumatic events as precipitating or exacerbating factors for sleep disturbances.

Several hypotheses have been posited to explain observed associations of poor sleep quality with a history of IPV victimization. Some authors have argued that heightened physiological arousal concomitant with psychological distress secondary to IPV victimization may contribute to poor sleep quality [38]. Available evidence supports this hypothesis as elevated corticotropin-releasing hormone (CRH) has been shown to lead to hyperactivity of the hypothalamic-pituitary-adrenal (HPA) axis, which is associated with sleep disturbances [39, 40]. Yet other investigators have suggested that traumatic brain injury attributable to IPV may account for observed associations of IPV victimization and altered sleep patterns. For instance, investigators have proposed that IPV-induced alterations in the amygdala, which is responsible for regulating mood and the medial prefrontal cortex, may lead to diminished coping skills, maladaptive behaviors and contribute to exacerbating sleep disturbances and poor sleep quality [41]. Additional research is warranted to more thoroughly examine these and other mechanisms hypothesized to account for observed associations of exposure to stressors, including IPV, with poor sleep quality.

Several limitations must be considered when interpreting the results of our study. First, intimate partner abuse was assessed based on self-report; and so we cannot rule out the influence of errors in recall of lifetime and prevalent IPV experiences. Ellsberg and colleagues have noted that study participants are likely to minimize experiences of past abuse rather than suggest that they had experienced abuse in their lifetime [42]. Hence, errors in maternal recall of IPV experiences in our study may have led to an underestimation of reported odds ratios. Furthermore, our use of well-trained interviewers who were blinded to specific study hypotheses and who used standard questionnaires to collect information from all study participants helped to mitigate the likelihood of systematic reporting errors in our study. Second, our use of self-reported data for subjective measures of sleep disturbances and other covariates may have introduced some degree of measurement error. Future studies that collect objective measures of sleep patterns and sleep quality (using polysomnography or actigraphy) may overcome concerns about these potential errors. Third, the effect of psychological abuse was not assessed in this study which may lead to an attenuation of the true association towards the null value. As a result, the magnitude of associations reported in our study tend to be conservative estimates. Finally, the participants in this study were pregnant women living in Lima, Peru and thus, the results may not be generalizable to other populations. The facts that our results are similar to those reported by others [36, 37], however, serve to mitigate these concerns.

Several strengths of our study merits discussion. First, our large sample size and the high prevalence of sleep disturbances, gave us sufficient statistical power to study the associations of interest. Second, we completed multivariable logistic regression analyses that accounted for potential confounding by sociodemographic factors; and we assessed the independent and joint contributions of IPV victimization and antepartum depression on odds of stress-related sleep disturbances and poor sleep quality. Lastly, our use of multiple measures for sleep disturbances, including our novel use of the Ford Insomnia Response to Stress Test to assess maternal vulnerability to stress-related sleep disturbance, extends the literature and recognizes the multi-faceted nature of sleep health among pregnant women.

The adverse effects of IPV on women’s physical, sexual, and reproductive and mental health have been well documented, although few have focused on sleep health an important correlate of physical and mental health. Our findings along with those of others [35–37] may be used by public health stakeholders including policy makers, public health administrators and clinicians to inform the development and implementation of effective interventions within the public health system for women who are victims of IPV. On the basis of our findings and those of others, we argue that effective interventions must take into consideration the influences of IPV victimization on women’s sleep health.

Addressing stress-related sleep disorders among pregnant victims of IPV in health care settings might enhance safety planning and prevent the development of maternal mental and physical health problems, as well adverse perinatal health outcomes that can arise from victimization. Preventive actions taken by obstetricians, public health and community health nurses, such as early identification, and appropriate first response or intervention, may be crucial for addressing the immediate and long-term effects of IPV. Of note, development and rigorous evaluation of targeted and cross-culturally acceptable trauma-informed care for pregnant women who have been victimized by physical and sexual violence is warranted.
